# Supplementary material for: The interactome of intact mitochondria by cross-linking mass spectrometry provides evidence for coexisting respiratory supercomplexes
Source: Mol Cell Proteomics. 2017 Dec 8;17(2):216–32. doi: 10.1074/mcp.RA117.000470 (PMC5795388; doi:10.1074/mcp.RA117.000470)
Supplement: Supplemental Data [file supp_RA117.000470_133922_0_supp_23978_fzffwf.zip › spectra_annotation/mito_DR_spectra_annotation/204-1-7-1-12-1.pdf]

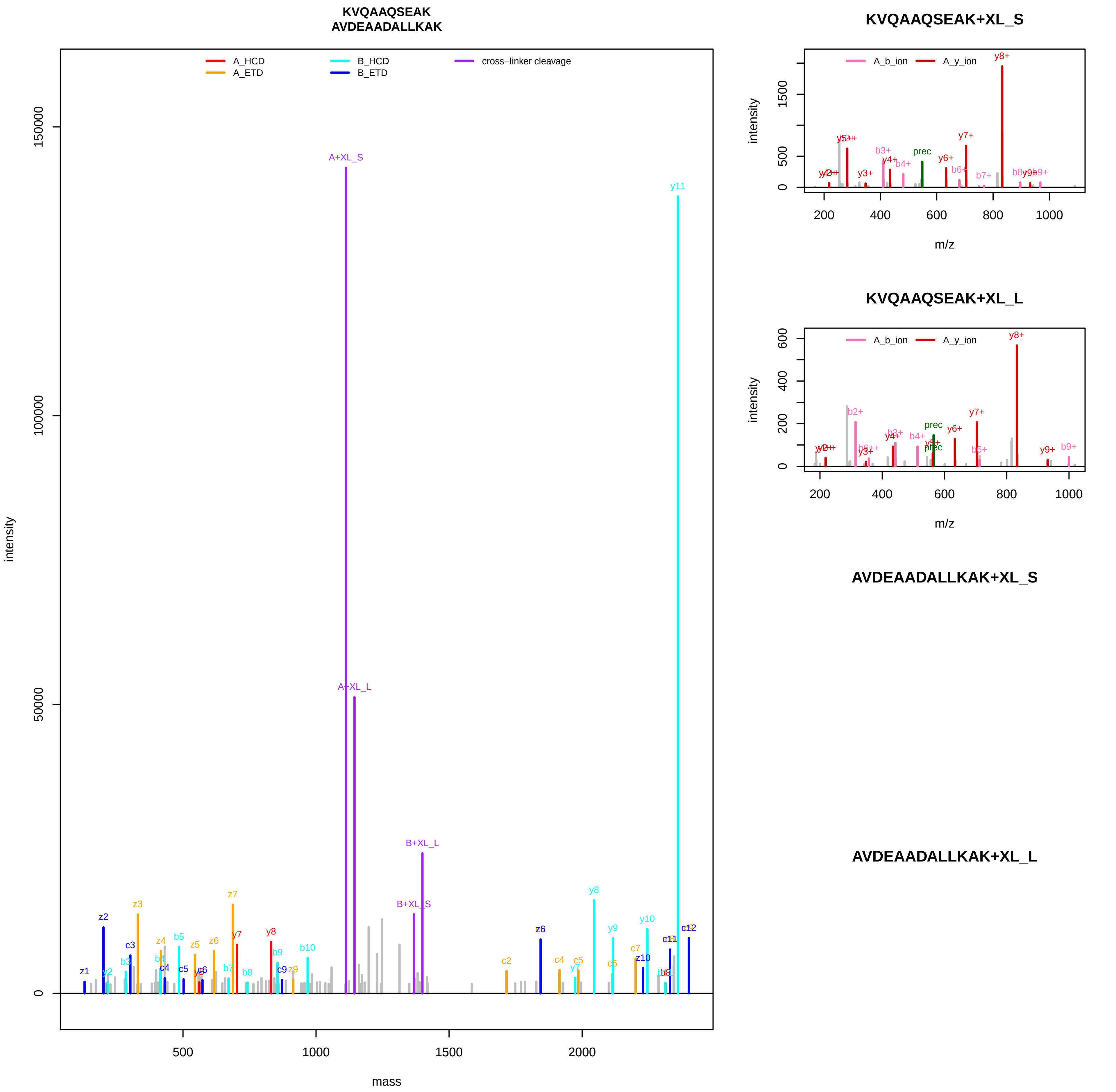

KVQAAQSEAK
AVDEAADALLKAK
A_HCD
A_ETD
B_HCD
B_ETD
cross−linker cleavage
intensity
mass
0
50000
100000
150000
500
1000
1500
2000
A+XL_S
A+XL_L
B+XL_L
B+XL_S
y11
y8
y9
y10
c12
c11
z6
z10
z2
z3
z7
y7
y8
b10
c3
z1
KVQAAQSEAK+XL_S
KVQAAQSEAK+XL_L
AVDEAADALLKAK+XL_S
AVDEAADALLKAK+XL_L
A_b_ion
A_y_ion
intensity
m/z
prec
y8+
y7+
y6+
y4+
y3+
b3+
b4+
b7+
y9+
b9+
b2+
